# Effects of probiotic supplementation with high-intensity interval training on cardiorespiratory endurance and metabolism in Middle-Aged Obese Women

**DOI:** 10.1080/15502783.2024.2425609

**Published:** 2024-12-01

**Authors:** Yi-Chen Chen, Hsuan-Yun Wang, Futoshi Ogita, Chi-Hsiang Hung, Chia-Hua Kuo, Jie-Ping Wang, Chia-Min Wang, Chien-Wen Hou, Ting-Yao Wang

**Affiliations:** a University of Taipei, Laboratory of Exercise Biochemistry, Institute of Sports Sciences, Taipei, Taiwan; b National Institute of Fitness and Sports in Kanoya, Department of Sports and Life Sciences, Kanoya, Japan; c Shih Hsin University, Office of Physical Education, Taipei, Taiwan; d University of Taipei, Department of Ball Sports, Taipei, Taiwan; e Hubei University, School of Physical Education, Wuhan, China; f Soochow University, Office of Physical Education, Taipei, Taiwan; g Center of Physical Education, Tzu Chi University,Hualien, Taiwan

**Keywords:** *Lactiplantibacillus plantarum* TWK10, gut microbiota, obese women, maximal oxygen uptake, time to exhaustion, running economy

## Abstract

**Introduction:**

High-intensity interval training (HIIT) has been shown to improve chronic diseases. Probiotics have been found to have similar effects. However, the additive effects of HIIT in combination with probiotics supplementation are unclear. The aim of current study was to investigate whether there were additive effects when implementing both HIIT and probiotics simultaneously.

**Methods:**

Forty-seven obese middle-aged women (Age: 44.5 ± 5.94 years, body fat percentage: 40.0 ± 4.1%) were recruited and assigned into four groups: control group (C, *n* = 12), probiotics group (P, *n* = 12), HIIT group (H, *n* = 11), and HIIT with probiotics group (HP, *n* = 12). All the participants consumed probiotics *(Lactiplantibacillus plantarum* TWK10, 6 × 10^10^ CFU/day) or placebo supplements daily. Exercise intervention groups conducted HIIT training (85–90% vVO_2_max for 2 min, followed by a 1-min inactive rest interval, repeated for 7 cycles) 3 sessions per week for 8 weeks. Anthropometry, cardiorespiratory endurance, blood glucose, and lipid profile were measured at baseline and after the 8-week intervention.

**Results:**

After the intervention, there were significant changes between groups in the variations and rates of change in waist circumference, hip circumference, and TTE. The waist circumference in group H significantly increased compared to groups C and P, while group HP did not show significant difference compared to group C. On the other hand, the hip circumference decreased significantly in group HP compared to group C, and the decreased rate in group HP was significantly greater than in groups C and P. Furthermore, the increase rates in TTE were higher in group H and HP compared to group C.

**Conclusion:**

HIIT improves TTE but negatively affects waist circumference compared to the control group. However, when combined with probiotics, the probiotics not only help enhance TTE but also counteract the negative impact on waist circumference and further reduce hip circumference, resulting in a synergistic effect.

**Clinical trial registration:**

ClinicalTrials.gov, identifier NCT06285578

## 1. Introduction

According to the World Health Organization, the top three causes of death worldwide in 2021, excluding COVID-19, were noninfectious diseases related to cardiopulmonary function [1]. Better cardiopulmonary fitness is associated with lower risk of cardiovascular disease (CVD) [2] and all-cause mortality [3]. Running economy (RE) is an important indicator for assessing aerobic endurance [4]. Maximal oxygen uptake (VO_2_max) can be considered as a gold indicator for assessing cardiorespiratory fitness [5]. On the other hand, the global obese populations are also growing rapidly. Obesity leads to chronic systemic inflammation, which is likely to induce metabolic disorders and ultimately increases overall mortality [6].

Women worldwide have a longer life expectancy than men [7]. In their middle age, they are at high-risk of developing obesity [8], threatening their long-term health [9]. Moreover, pregnancy-related conditions are risk factors for CVD as well [10]. Therefore, the improvement of cardiopulmonary fitness and the prevention of metabolic disorder in the middle-aged women are important issues to enhance their quality of life.

Lack of exercise is the main cause of most chronic diseases [3]. “Lack of time” is the main reason why people do not exercise regularly [11]. High-intensity interval training (HIIT) is a time-efficiency exercise mode, which takes less total time, making participants more willing to continue exercising [11]. Although time is short, there are great exercise benefits, such as reducing body fat, fasting blood insulin concentrations [12], arterial stiffness [13], and also contributing to improve RE [14]. For overweight and obese women, HIIT has been shown to reduce body fat and increase cardiorespiratory fitness [15]. On the other hand, gut microbiota affects metabolism as well. There are at least 10^14^ bacteria in the human intestine, and their genome is approximately 150-fold larger than the human genome [16]. Daily consumption of probiotic-fermented milk for 12 weeks helps reduce abdominal visceral fat, body weight, body mass index (BMI), waist, and hip circumference in overweight and obese men and women adults [17]. However, another study reported inconsistent results, showing that probiotics did not lead to improvements in body weight, BMI, or waist circumference in overweight and obese premenopausal women [18]. *Lactiplantibacillus plantarum* (former *Lactobacillus plantarum*), a symbiotic bacterium of humans, has been found to play a role in protecting intestinal health, improving inflammation and insulin sensitivity, and reducing the risk of CVD [19]. *Lactiplantibacillus plantarum* TWK10 (TWK10), a probiotic bacterium isolated from Taiwanese pickled vegetables, has been demonstrated to improve endurance exercise performance in animal study [20] and male adults [21]. Moreover, its potential for reducing body fat has been demonstrated in young male and female adults [22]. However, the effects of TWK10 in middle-aged obese women are still unclear.

Several studies have demonstrated the effect of HIIT or probiotic supplementation on improving aerobic capacity, body composition, and metabolic status. However, few studies have investigated whether they have a multiplier effect in middle-aged women. Previous animal study investigated the effect when combining HIIT and probiotics intervention and indicated the benefits of improving body weight and visceral fat gain caused by high-fat diets [23]. Moreover, 8 weeks of HIIT and probiotic intervention improves body weight, waist circumference, visceral fat, and insulin resistance in ovariectomized rats [24]. However, the effects in middle-age obese women remain unclear. Therefore, the purpose of this study was to investigate the effects of HIIT in combination with probiotic supplementation on anthropometry, cardiorespiratory fitness, and metabolic markers in middle-aged obese women.

## 2. Materials and methods

### 2.1. Participants and study design

Forty-seven obese women aged 35–55 years with no exercise habits were recruited. Since body fat may be excessive even with a normal BMI, the standard for obese women in the present study was a percentage of total body fat greater than 35% [25]. Given that the participants were obese middle-aged adults, it was challenging to control for the absence of medications associated with chronic conditions (e.g. those related to blood pressure or blood glucose regulation). However, any supplements or medications that could have directly interfered with the study intervention, such as additional probiotic powders or cold and flu medications containing antibiotics, were excluded if used within the three months prior to the start of the study or during the study. All participants were informed of the experimental procedures and signed an informed consent form that was approved by the Institutional Review Board of Taiwan Adventist Hospital. Prior to the start of the intervention, all participants were asked to maintain their usual diet and not to take additional probiotic supplements or medications containing antibiotics.

The study was placebo-controlled and double-blind trial. All participants were assigned into four groups: (1) placebo control group (C, *n* = 12); (2) probiotic group (P, *n* = 12); (3) HIIT group (H, *n* = 11), and (4) HIIT with probiotic group (HP, *n* = 12). Participants in all groups consumed probiotic supplements or placebo daily before breakfast and those in the exercise groups participated in a self-monitored HIIT training for 3 sessions per week for 8 weeks. Participants were given probiotics or placebo in identical-looking packaging. The researchers who gave the supplement to the participants and conducted the study were different people from the administrators who dispensed the supplement. The participants and researchers were unaware of the contents of the supplements to which each participant was supplemented until the study was over. Three major tests were conducted at baseline and post-intervention, within one week before or after the intervention: anthropometry measurement, cardiorespiratory endurance, and blood glucose and lipid profile. Since all participants were office workers, it was not feasible to conduct all measurements at a fixed time in the morning. Consequently, the pretest and posttest for anthropometry and cardiorespiratory endurance were conducted after work, between approximately 17:00 and 18:00. On the day of the test, participants were instructed to refrain from consuming any food after lunch (12:00-13:00) and before the testing session. Blood sampling, however, was performed between 7:30 and 8:30 a.m., following an 8-hour fast, prior to the participants starting their workday. To assess the dietary intake among the groups, three-day dietary food records (comprised 2 weekdays and 1 weekend day) were administered before and after the intervention, and their caloric intake and dietary content were assessed by a dietitian.

### 2.2. Measurement

#### 2.2.1. Anthropometry

Dual-energy X-ray absorptiometry (DEXA) (Lunar iDXA, GE Medical Systems, WI, USA) was used to measure body fat percentage and other body composition parameters. Height (minimum unit 1 mm) and body weight (minimum unit 0.1 kg) was measured with an electronic scale. Body mass index (BMI) was calculated by the formula: weight (kg)/height (m^2^). Waist circumference was measured at the middle between the lower edge of the trunk rib to the upper edge of the ilium and hip measurement was measured at the maximum hip circumference using a measuring tape (minimum unit 1 mm) [26]. Body fat percentage, fat mass, muscle mass, and bone mineral density (BMD) were originally important indicators in current study by using DEXA. However, the machine malfunctioned during the posttest, resulting in abnormal and uncorrectable data accuracy. Therefore, the posttest values of body composition were not used. However, according to a previous study, hip circumference is positively correlated with body fat and abdominal obesity in menopausal women [27]. Moreover, there is a strong correlation between the amount of body fat calculated from hip circumference and the amount of body fat detected by DEXA [27]. Therefore, the estimation of obesity by hip circumference might have a great credibility.

#### 2.2.2. Cardiorespiratory endurance

Incremental intensity treadmill exercise test was conducted using T645L treadmill (SportsArt, Tainan, Taiwan), and the design principle was based on previous study [28]. The starting speed of the test was 5.4 km/h with the incline 0%. The speed was increased by 1.8 km/h every 3 minutes. When the running speed was increased to 9.0 km/h, the participants ran at this speed for 3 minutes and then increased the incline by 2% every minute until the participants were exhausted. The researcher observed the condition of the participants throughout the test and helped stop the treadmill when necessary. Oxygen uptake was measured with an energy metabolism analyzer (MetaMax 3B, Cortex, Germany) throughout the test. After the test, VO_2_max and time to exhaustion (TTE) were collected and recorded. The formula referred to Mooses et al. [29] was used to calculate the running economy (RE) values by using the maximum running speed of each participant before increasing the incline and the oxygen uptake at a fixed speed. The formula is as follows:

(1) $$\mathrm{RE}\;\left(\mathrm{ml}/\mathrm{kg}/\mathrm{km}\right)1000\times VO_{2}\left(\mathrm{mL}/\min/\mathrm{kg}\right)\times \mathrm{speed}\;{\left(m/min\right)}^{-1}$$

#### 2.2.3. Blood glucose and lipid profile

Fasting blood glucose (FBG), total cholesterol (TC), triglyceride (TG), high-density lipoprotein cholesterol (HDL-C), and low-density lipoprotein cholesterol (LDL-C) concentrations were measured by blood samples. All participants fasted for 8 hours before blood collection and blood samples were taken from cephalic vein at the Health Examination Center of Taiwan Adventist Hospital in the morning of the prescribed time. The five metabolic markers were analyzed by colorimetric method using an automated biochemical analyzer (UniCel DxC 800 Synchron, Beckman coulter, Brea, California, USA).

### 2.3. Intervention

#### 2.3.1. HIIT

After completing the incremental intensity test, the velocity at VO_2_max (vVO_2_max) was recorded by substituting the individual’s maximum oxygen uptake into the equation through the linear relationship between exercise intensity and oxygen uptake [30], which was used as the basis for setting the intensity during subsequent HIIT. The HIIT protocol referred to Wang et al. [31] and set the training intensity as 85–90% VO_2_max. After warming up 3 minutes on treadmill at 60% vVO_2_max, the participants ran at a speed of 85–90% vVO_2_max for 2 minutes, followed by a 1-minute inactive rest interval, repeated for a total of 7 cycles. After completing the intervals, participants performed a 1-minute recovery at 3.6 km/h. Participants were asked to complete 3 hIIT sessions per week for 8 weeks for a total of 24 training sessions. The intensity and interval structure of the HIIT training were consistent throughout the 8-week intervention period.

#### 2.3.2. Probiotics

*Lactiplantibacillus plantarum* TWK10 (TWK10), a strain of probiotics isolated from Taiwanese-pickled vegetables [20,21], was used as the probiotic supplement. The probiotics were packaged in powder form and each packet contained 6 × 10^10^ CFU of TWK10 strain (SYNBIO TECH, Kaohsiung, Taiwan). The placebos were also packaged in powder form with yeast powder replacing TWK10 to reduce the taste difference between placebo and probiotic. The same fixed ingredients were added to the packaging of both probiotics and placebos to facilitate shaping and increase palatability. Each participant consumed one packet of probiotic or placebo every morning on a fasting status for 8 weeks.

### 2.4. Data analysis and statistics

All data are presented as median with interquartile range (IQR). The *p*-values <0.05 were considered statistically significant. Shapiro–Wilk test was used to check the distribution of the variables. Since some data did not follow a normal distribution, non-parametric validation was used uniformly. Kruskal – Wallis test was used to analyze the differences in baseline, variations, and rates of change between groups. If there was a significant difference, Mann–Whitney U test was used to detect the difference between two groups. Wilcoxon signed-rank test was used to analyze the differences within group. The effect sizes between two values were calculated (Cohen’s effect size, *d*).

## 3. Results

There were no statistical differences in the baseline data among groups (Table 1). One participant in the HP group reported mild knee pain after the experiment, but no pain was experienced during any of the HIIT sessions or measurements. All participants tolerated both the HIIT protocol and probiotic supplementation and completed the intervention.

**Table 1.** Baseline of four groups.

	C	P	H	HP	*p*
Age (years)	40.3 (39.3−43.9)	42.7 (40.7−47.5)	43.1 (38.0−53.3)	45.6 (42.0−50.2)	0.419
Height (cm)	160.5 (154.4−162.2)	157.5 (154.3−158.3)	160.4 (157.1−163.2)	157.2 (153.9−162.9)	0.503
Body weight (kg)	64.8 (58.1−70.8)	64.2 (60.8−69.1)	63.5 (60.1−67.2)	62.7 (52.9−67.0)	0.838
BMI (kg/m^2^)	25.3 (23.8−28.2)	26.1 (24.6−27.4)	24.7 (23.2−25.5)	24.0 (22.0−26.0)	0.358
Body fat (%)	40.4 (38.4−43.2)	41.1 (39.1−43.4)	39.8 (36.9−43.1)	38.3 (35.4−41.7)	0.568
Fat mass (kg)	25.9 (24.1−28.9)	26.0 (23.0−29.1)	25.5 (21.1−27.2)	23.5 (19.4−26.1)	0.515
Muscle mass (kg)	36.7 (34.3−39.1)	36.5 (34.9−38.4)	37.0 (34.5−40.1)	36.9 (33.1−39.5)	0.992
BMD (g/cm^2^)	1.1 (1.1−1.2)	1.1 (1.1−1.2)	1.1 (1.0−1.2)	1.1 (1.1−1.2)	0.496
Waist (cm)	85.5 (83.8−89.8)	86.5 (83.5−94.5)	80.5 (77.0−85.0)	82.5 (72.0−84.3)	0.101
Hip (cm)	97.0 (95.0−99.3)	99.5 (94.8−102.5)	97.0 (94.0−99.0)	98.8 (91.0−101.3)	0.716
VO_2_max (mL/min^/^kg)	32.0 (30.5−35.0)	33.0 (29.0−34.0)	36.0 (33.5−37.5)	34.0 (28.0−36.0)	0.153
TTE(s)	480.0 (370.0−600.0)	400.0 (340.0−460.0)	570.0 (525.0−605.0)	480.0 (345.0−585.0)	0.055
RE (ml/kg/km)	210.7 (198.0−222.2)	216.7 (209.2−221.7)	208.7 (201.7−227.1)	220.0 (205.0−228.2)	0.949
FBG (mg/dL)	94.0 (90.0−100.0)	93.0 (88.5−102.5)	88.5 (85.0−89.0)	93.0 (87.5−96.0)	0.137
TC (mg/dL)	191.0 (170.0−206.0)	181.0 (165.5−195.5)	189.5 (175.0−211.8)	185.0 (176.0−202.0)	0.799
TG (mg/dL)	86.0 (74.0−138.0)	120.0 (90.5−167.5)	100.0 (79.5−164.8)	75.0 (70.5−115.5)	0.410
HDL-C (mg/dL)	52.0 (43.0−62.5)	44.0 (38.0−49.5)	54.5 (44.8−62.0)	56.0 (49.5−60.0)	0.093
LDL-C (mg/dL)	117.1 (102.5−130.8)	126.5 (104.6−130.9)	116.2 (101.4−139.3)	111.7 (100.8−136.7)	0.985

Values are presented as median with IQR. C: placebo control group; P: probiotic group; H: HIIT group; HP: HIIT with probiotic group. *P*-values represent intergroup differences.

**Figure 1.**
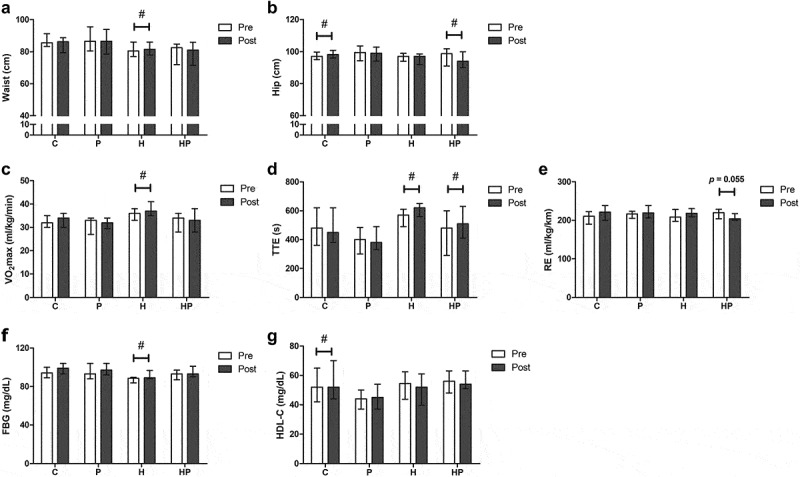
The effects of probiotics and HIIT on (a) waist circumference, (b) hip circumference, (c) VO_2_max, (d) TTE, (e) RE, (f) FBG, and (g) HDL-C in each group. Values are presented as raw data (median and IQR). ^#^Significant difference between pre and post-test within group, *p* < 0.05.

At baseline and after the 8-week intervention, there were no significant differences in daily caloric intake or dietary content between the four groups. However, post-intervention caloric intake in the HP group was significantly lower compared to baseline (Table 2). Neither body weight nor BMI was affected by probiotics and HIIT. Waist circumference increased significantly in group H (80.5 [77.0–85.0] to 81.5 [78.5–85.5], *p* = 0.030, *d* = 0.19), while hip circumference increased significantly in group C (97.0 [95.0–99.3] to 98.3 [96.0–100.3], *p* = 0.037, *d* = 0.38) and decreased significantly in group HP (98.8 [91.0–101.3] to 94.0 [90.0–99.6], *p* = 0.035, *d* = 0.25) (Figure 1a,b). VO_2_max increased significantly in group H (36.0 [33.5–37.5] to 37.0 [35.0–39.0], *p* = 0.015, *d* = 0.53) (Figure 1c). TTE significantly increased in group H (570.0 [525.0–605.0] to 620.0 [580.0–640.0], *p* = 0.026, *d* = 0.45) and HP (480.0 [345.0–585.0] to 510.0 [415.0–610.0], *p* = 0.032, *d* = 0.37) (Figure 1d). There was a trend toward improved RE in group HP (220.0 [205.0–228.2] to 204.0 [200.6–213.3], *p* = 0.055, *d* = 0.60) (Figure 1e). FBG increased significantly in group H (88.5 [85.0–89.0] to 89.0 [88.3–94.3], *p* = 0.029, *d* = 0.86) (Figure 1f) and HDL-C in group C was significantly higher in posttest (52.0 [43.0–62.5] to 52.0 [45.0–69.0], *p* = 0.046, *d* = 0.23) (Figure 1g). TC, TG, and LDL-C did not show any significant difference.

**Table 2.** Daily dietary intake and content.

	C	P	H	HP	*p*
Baseline					
Calories (kcal)	1738.0 (1263.0−2133.0)	1596.5 (1506.8−1723.0)	1610.0 (1431.0−1815.5)	1853.0 (1657.5−1906.3)	0.798
CHO (g)	185.3 (168.3−234.9)	177.4 (163.4−193.9)	186.1 (161.8−212.5)	220.7 (174.3−249.2)	0.389
Protein (g)	77.4 (43.5−82.3)	56.4 (52.8−76.7)	65.6 (59.7−72.4)	67.3 (57.8−81.6)	0.899
Fat (g)	76.1 (55.5−95.0)	72.6 (69.3−83.9)	74.0 (67.1−77.7)	74.5 (69.2−81.1)	0.894
Fiber (g)	10.0 (9.6−10.8)	9.8 (7.3−14.9)	10.8 (10.4−16.1)	12.6 (9.5−15.3)	0.611
Post−intervention					
Calories (kcal)	1524.5 (1366.0−1745.5)	1504.0 (1233.5−1603.5)	1636.0 (1543.0−1675.0)	1626.0^#^ (1463.5−1795.0)	0.302
CHO (g)	174.9 (153.8−198.6)	160.8 (142.6−176.7)	175.6 (165.1−188.5)	190.7 (145.7−205.6)	0.672
Protein (g)	54.1 (48.4−64.8)	57.1 (47.7−63.5)	67.5 (60.1−72.5)	61.7 (56.2−74.4)	0.153
Fat (g)	70.4 (57.9−82.3)	63.9 (51.3−71.5)	73.2 (72.4−74.6)	69.4 (57.5−82.3)	0.478
Fiber (g)	8.2 (5.3−12.7)	10.2 (8.0−13.7)	12.7 (11.4−13.3)	10.6 (6.6−16.8)	0.704

Values are presented as median with IQR. C: placebo control group; P: probiotic group; H: HIIT group; HP: HIIT with probiotic group; CHO: Carbohydrate. *P*-values represent intergroup differences. ^#^Significant difference between pre and posttest within HP group, *p* = 0.041.

**Figure 2.**
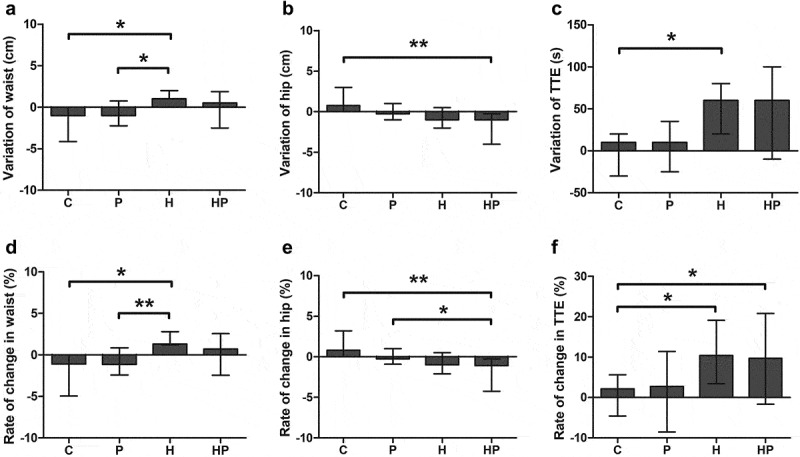
The variation and rate of change in (a&d) waist circumference, (b&e) hip circumference, and (c&f) TTE in each group. Values are presented as raw data (median and IQR). *Significant different between groups, *p* < 0.05; **, *p* < 0.01.

There were significant changes between groups in the variations and rates of change in waist circumference, hip circumference, and TTE. For waist circumference, the variation in group H (1.0 [1.0 to 2.0]) was significantly higher than in group C (−1.0 [−2.4 to 0.0], *p* = 0.016, *d* = 0.92) and P (−1.0 [−1.8 to 0.3], *p* = 0.019, *d* = 0.87), and the rate of change were significantly higher in group H (1.3 [1.2 to 2.7]%) than in group C (−1.1 [−2.8 to 0.0]%, *p* = 0.013, *d* = 0.96) and P (−1.2 [−2.1 to 0.3]%, *p* = 0.009, *d* = 1.02) (Figure 2a,d). For hip circumference, the variation in group HP (−1.0 [−4.0 to −0.8]) was significantly lower than that in group C (0.8 [0.0 to 3.0], *p* = 0.004, *d* = 1.31), and the rate of change in group HP (−1.1 [−4.0 to −0.7]%) was significantly lower than that in group C (0.8 [0.0 to 3.1]%, *p* = 0.005, *d* = 1.27) and P (−0.3 [−0.8 to 1.0]%, *p* = 0.020, *d* = 0.80) (Figure 2b,e). For TTE, the variation in group H (60.0 [35.0 to 80.0]) were significantly increased compared with group C (10.0 [−20.0 to 20.0], *p* = 0.010, *d* = 0.95), and the rates of change in group H (10.4 [5.9 to 14.9]%, *p* = 0.013, *d* = 0.98) and HP (9.7 [2.8 to 20.4]%, *p* = 0.028, *d* = 1.02) were significantly higher than that in group C (2.1 [−3.8 to 5.2]%) (Figure 2c,f).

## 4. Discussion

### 4.1. Anthropometry

A previous study has pointed out that HIIT is beneficial to reduce body weight and waist circumference [32], which does not match the result of the present study. Martins et al. [32] demonstrated that HIIT training 3 sessions per week for 12 weeks at 85–90% of maximal heart rate reduced waist and hip circumference in obese individuals, while HIIT of the same intensity, frequency, and total number of weeks with half the training time of a single session (1/2 HIIT) achieved similar metabolic effects. Another study indicated that for obese individuals, although HIIT for less than 12 weeks can increase VO_2_max, only doing HIIT for more than 12 weeks can improve waist circumference [33]. These results show that the factor in reducing waist circumference with HIIT may not be the duration of a single session, but the continuity of the exercise. Only 8 weeks of HIIT in the current study may not be enough to achieve the effect of waist circumference reduction.

In terms of probiotics, currently probiotics are inconsistent in improving obesity. In a previous study, healthy men and women who consumed *L. plantarum* TWK10 (TWK10) for 6 weeks showed no change in BMI but a reduction in body fat [22]. No change in BMI can be inferred from the stable body weight. However, the body compositions were improved. Although it is unfortunate that posttest body composition values were not available in the present study, since it had been reported that there is a strong correlation between the amount of body fat calculated from hip circumference and the amount of body fat detected by DEXA [27], it is speculated that body fat loss might occur in the HP group based on the reduction in hip circumference. On the other hand, a previous study showed that 6 weeks of TWK10 supplementation reduced body weight in mice [20], which is inconsistent with the present study. The difference between the results of the studies may be due to the difference in species between mice and humans. Although the intestinal flora of mice is similar to that of humans, the diversity and complexity of the human intestinal flora are higher than that of mice [34], which makes it more difficult to alter the composition of the human intestinal flora. In addition, compared to mice living under a fixed diet and environment, human beings have many variations in work and rest, diet, environment, and stress that may affect the composition of intestinal flora [35,36].

Climate change is also an important factor affecting body weight and waist circumference. Our study was unintentionally conducted between October and January of the following year, which previous study has indicated to be a period of greater weight gain in adults [37]. This phenomenon may be caused by changes in the secretion of appetite hormones. Tomasik et al. [38] demonstrated that a lower temperature stimulates the secretion of ghrelin, which stimulates the hunger center of the brain and promotes appetite. Moreover, exercising at a lower temperature significantly increases energy intake after exercise [39]. This may be due to the reasons mentioned above, offsetting the benefits of HIIT in reducing body weight and even increase waist circumference in the present study. However, when HIIT is combined with probiotic supplementation, the increase in waist circumference can be avoided. A previous study has indicated that *L. plantarum* TWK10 can help with weight control when dietary intake is significantly increased [20]. On the other hand, dietary content affects the component of intestinal flora [36], indicating that diet may influence the effects of probiotic supplementation. In our study, although we asked the subjects to maintain their usual eating habits and assessed their daily caloric intake before and after the intervention, there was no strict control or monitoring of daily dietary content during the intervention period, which may affect the study results.

### 4.2. Cardiorespiratory endurance

Many studies have indicated that HIIT is effective in increasing VO_2_max, improving cardiorespiratory fitness [33,40], and increasing TTE [41], which is consistent with the current study.

Previous human studies have shown that TWK10 supplementation can significantly increase running TTE [21,22]. However, in the present study, only group HP showed a significant increase in TTE, while group P showed no such benefit. This could be due to the insufficient dose of probiotics and the age gap between the subjects. In the two previous studies, 1 × 10^11^ CFU [21] and 3 × 10^11^ CFU [22] of TWK10 strain per day were supplemented, while the present study only supplemented 6 × 10^10^ CFU per day. In addition, the subjects in the previous studies were around 20–40 years old, whereas in the present study were 35–55 years old. According to a previous study, the diversity of intestinal flora increases with age [42], which makes the composition of intestinal flora more difficult to change. Therefore, it is possible that disparities in the dose of probiotic supplementation and gut microbial diversity could lead to inconsistencies between the current findings and previous study.

In the present study, a trend toward improvement in RE was found when HIIT and probiotic were intervened simultaneously. The lower the RE value, the less oxygen is consumed at a steady intensity, and the more efficient the oxygen utilization is [4]. In trained runners, RE may be different even if VO_2_max is similar, and those with better RE have better performance, which could also demonstrate that RE is more predictive of exercise performance than VO_2_max [43]. Although there are currently few studies exploring whether probiotics can improve RE, some studies have mentioned that the composition of intestinal flora is related to mitochondrial function, affecting ATP production [44]. In addition, an animal study found that TWK10 helps increase the number of type I muscle fibers and reduce lactate, creatine kinase, and other indicators related to muscle fatigue [20]. Therefore, it is possible that TWK10 helps to improve muscle energy utilization efficiency and aerobic endurance. Although the results of RE in the current study did not achieve a significant difference, it is possible that increasing the dosage of probiotics or the duration of the intervention may lead to different results. Further studies are needed to investigate in this area.

### 4.3. Blood glucose and lipid profile

It is worth discussing that even though FBG was elevated in H group, the elevated value of 89.0 (88.3–94.3) mg/dL was still normal according to the American Diabetes Association’s diagnostic criteria for diabetes [45]. However, in the present study, it was indisputable that HIIT increased FBG level. A previous study showed that doing HIIT 3 times per week for 12 weeks could improve FBG and HDL-C [40], which is inconsistent with the results of the present study. However, in the previous study, the HIIT was conducted in a progressive manner, starting with moderate intensity continuous training and gradually increasing the intensity to 85% VO_2_peak HIIT. In the present study, the intensity was directly increased to 85–90% VO_2_max in the first training. For those who were not used to exercise, such high intensity may cause extreme stress and inflammation. Previous studies have pointed out that high-intensity exercise increases gastrointestinal permeability [46] and the production of endotoxin (Lipopolysaccharide, LPS) [47]. Endotoxin is present on the outer membrane of Gram-negative bacteria and can be used as an indicator to assess the health status of the intestinal mucosa [48]. Under normal conditions, the intestinal mucosal epithelial cells are tightly connected so that external bacteria, drugs, food, or toxins cannot easily pass through the intestinal mucosa. When the intestinal permeability increases (also known as leaky gut), endotoxin can cross the intestine mucosa easily and enter the blood circulation [48,49], causing a systemic inflammatory response, and even leading to the development of endotoxemia or sepsis [50]. Previous studies indicated that blood endotoxin levels are higher in diabetic patients than healthy individuals, and blood endotoxin concentrations are positively correlated with blood glucose fluctuations [49] and FBG and are negatively correlated with HDL-C [51]. The probiotic *L. plantarum* helps to maintain the integrity of the intestinal mucosa, preventing the occurrence of leaky gut [52]. The negative effects on FBG caused by HIIT in the present study may be due to the inflammatory response. However, it can be offset when HIIT in combination with probiotics. This may be a benefit of probiotics in helping to stabilize the intestinal mucosa barrier and further improve inflammation. However, the causal relationship is still unclear and future studies are needed to investigate the relevant mechanisms.

On the other hand, the blood HDL-C levels fluctuate with the menstrual cycle, with the lowest concentration during the menstrual period, the highest concentration before ovulation, and gradually decreases after ovulation, with a difference of about 3.5 mg/dL [53]. Additionally, blood HDL-C levels gradually increase before menopause and decrease after menopause [54]. The present study did not standardize the menstrual cycle and menopausal status of each subject, which might be a limitation of the study.

### 4.4. Strengths and limitations

Currently, the combined effects of HIIT and probiotics in middle-aged obese women are not fully known. The present study explored the effects of simultaneous intervention of both, which could be beneficial for future research and applications. Nevertheless, there are some limitations need to be noted and improved. Although we were unaware in advance that the season affects body weight, previous studies have pointed out this fact, and therefore future studies should take season into account. Moreover, the lack of performing dietary control and the lack of standardization of each subject’s menstrual cycle during the intervention period may have affected the results. The effect of the menstrual cycle and menopausal status should be considered when conducting studies in women.

## 5. Conclusions

HIIT helps to improve TTE but causes a negative effect on waist circumference in middle-aged obese women. When HIIT in combination with probiotics, probiotics help not only enhance TTE but also counteract the negative impact on waist circumference and further reduce hip circumference, resulting in a synergistic effect. On the other hand, based on the within-group comparison, although HIIT improves VO_2_max, it seems to have a negative impact on FBG. However, probiotics help ameliorate this negative effect. In the perspectives obtained from the present study, while HIIT brings benefits to the human body, high-intensity exercise may also cause slight negative effects that probiotics might help mitigate. Further studies are needed to investigate the relevant mechanisms, and the limitations of the present study should be avoided.

## References

[1] World Health Organization. The top 10 causes of death. [2024 Aug 7]. Available from: https://www.who.int/news-room/fact-sheets/detail/the-top-10-causes-of-death

[2] Fernstrom M, Fernberg U, Eliason G, et al. Aerobic fitness is associated with low cardiovascular disease risk: the impact of lifestyle on early risk factors for atherosclerosis in young healthy Swedish individuals - the lifestyle, biomarker, and atherosclerosis study. Vasc Health Risk Manag. 2017;13:91–15. doi: 10.2147/VHRM.S125966 28352184 PMC5358957

[3] Booth FW, Roberts CK, Laye MJ. Lack of exercise is a major cause of chronic diseases. Compr Physiol. 2012;2(2):1143–1211. doi: 10.1002/cphy.c110025 23798298 PMC4241367

[4] Barnes KR, Kilding AE. Running economy: measurement, norms, and determining factors. Sports Med - Open. 2015 Dec;1(1):8. doi: 10.1186/s40798-015-0007-y 27747844 PMC4555089

[5] Hill AV, Long CNH, Lupton H. Muscular exercise, lactic acid, and the supply and utilisation of oxygen.—parts IV-VI. R Soc Of Lond Ser B. 1924;97(681):84–138. doi: 10.1098/rspb.1924.0045

[6] Abdelaal M, le Roux Cw, Docherty NG, et al. Morbidity and mortality associated with obesity. Ann Transl Med. 2017 Apr;5(7):161. doi: 10.21037/atm.2017.03.107 28480197 PMC5401682

[7] Thornton J. WHO report shows that women outlive men worldwide. BMJ. [2019 Apr 5];365:l1631. doi: 10.1136/bmj.l1631 30952650

[8] Au N, Hauck K, Hollingsworth B. Employment, work hours and weight gain among middle-aged women. Int J Obes (Lond). 2013 May;37(5):718–724. doi: 10.1038/ijo.2012.92 22710930

[9] Moreira MA, Vafaei A, da Camara SMA, et al. Metabolic syndrome (MetS) and associated factors in middle-aged women: a cross-sectional study in Northeast Brazil. Women Health. 2019 Nov;15:1–17. doi: 10.1080/03630242.2019.1688445 31726939

[10] Leonard EA, Marshall RJ. Cardiovascular disease in women. Prim Care. 2018 Mar;45(1):131–141. doi: 10.1016/j.pop.2017.10.004 29406939

[11] Gibala MJ. High-intensity interval training: a time-efficient strategy for health promotion? Curr Sports Med Rep. 2007;6(4):211–213. doi: 10.1097/01.CSMR.0000306472.95337.e9 17617995

[12] Trapp EG, Chisholm DJ, Freund J, et al. The effects of high-intensity intermittent exercise training on fat loss and fasting insulin levels of young women. Int J Obes (Lond). 2008 Apr;32(4):684–691. doi: 10.1038/sj.ijo.0803781 18197184

[13] Hasegawa N, Fujie S, Horii N, et al. Effects of different exercise modes on arterial stiffness and nitric oxide synthesis. Med Sci Sports Exerc. 2018 Jun;50(6):1177–1185. doi: 10.1249/MSS.0000000000001567 29381650

[14] Zatoń M, Michalik K. Effects of interval training-based glycolytic capacity on physical fitness in recreational long-distance runners. Hum Mov. 2015;16(2):71–77. doi: 10.1515/humo-2015-0029

[15] Guo L, Chen J, Yuan W. The effect of HIIT on body composition, cardiovascular fitness, psychological well-being, and executive function of overweight/obese female young adults. Front Psychol. 2022;13:1095328. doi: 10.3389/fpsyg.2022.1095328 36743598 PMC9891140

[16] Tilg H, Kaser A. Gut microbiome, obesity, and metabolic dysfunction. J Clin Invest. 2011;121(6):2126–2132. doi: 10.1172/JCI58109 21633181 PMC3104783

[17] Kadooka Y, Sato M, Imaizumi K, et al. Regulation of abdominal adiposity by probiotics (Lactobacillus gasseri SBT2055) in adults with obese tendencies in a randomized controlled trial. Eur J Clin Nutr. 2010 Jun;64(6):636–643. doi: 10.1038/ejcn.2010.19 20216555

[18] Madjd A, Taylor MA, Mousavi N, et al. Comparison of the effect of daily consumption of probiotic compared with low-fat conventional yogurt on weight loss in healthy obese women following an energy-restricted diet: a randomized controlled trial. Am J Clin Nutr. 2016 Feb;103(2):323–329. doi: 10.3945/ajcn.115.120170 26702123

[19] Kasselman LJ, Vernice NA, DeLeon J, et al. The gut microbiome and elevated cardiovascular risk in obesity and autoimmunity. Atherosclerosis. 2018 Apr;271:203–213. doi: 10.1016/j.atherosclerosis.2018.02.036 29524863

[20] Chen YM, Wei L, Chiu YS, et al. Lactobacillus plantarum TWK10 supplementation improves exercise performance and increases muscle mass in mice. Nutrients. [2016 Apr 7];8(4):205. doi: 10.3390/nu8040205 27070637 PMC4848674

[21] Huang WC, Hsu YJ, Li H, et al. Effect of lactobacillus plantarum TWK10 on improving endurance performance in humans. Chin J Physiol. 2018 Jun;61(3):163–170. doi: 10.4077/CJP.2018.BAH587 29962176

[22] Lee CC, Liao YC, Lee MC, et al. Different impacts of heat-killed and viable Lactiplantibacillus plantarum TWK10 on exercise performance, fatigue, body composition, and gut microbiota in humans. Microorganisms. [2022 Nov 3];10(11):2181. doi: 10.3390/microorganisms10112181 36363775 PMC9692508

[23] Foroozan P, Koushkie Jahromi M, Nemati J, et al. Probiotic supplementation and high-intensity interval training modify anxiety-like behaviors and corticosterone in high-fat diet-induced obesity mice. Nutrients. [2021 May 21];13(6):1762. doi: 10.3390/nu13061762 34064242 PMC8224367

[24] Bayat Z, Damirchi A, Hasannejad-Bibalan M, et al. Metabotropic effect of probiotic supplementation and high-intensity interval training in menopause-induced metabolic syndrome in rats. J Menopausal Med. 2023 Apr;29(1):29–39. doi: 10.6118/jmm.22037 37160300 PMC10183765

[25] World Health Organization. Physical status: the use and interpretation of anthropometry. Vol. 854. Geneva: World Health Organization; 1995. p. 1–452. WHO Technical Report Series. 8594834

[26] World Health Organization. WHO STEPS surveillance manual: the WHO STEPwise approach to noncommunicable disease risk factor surveillance. Geneva: World Health Organization; 2017.

[27] Raja C, Hansen R, Baber R, et al. Hip girth as a predictor of abdominal adiposity in postmenopausal women. Nutrition. 2004 Sep;20(9):772–777. doi: 10.1016/j.nut.2004.05.007 15325686

[28] Bentley DJ, Newell J, Bishop D. Incremental exercise test design and analysis: implications for performance diagnostics in endurance athletes. Sports Med. 2007;37(7):575–586. doi: 10.2165/00007256-200737070-00002 17595153

[29] Mooses M, Tippi B, Mooses K, et al. Better economy in field running than on the treadmill: evidence from high-level distance runners. Biol Sport. 2015 Jun;32(2):155–159. doi: 10.5604/20831862.1144418 26060340 PMC4447762

[30] Morgan D, Baldini FD, Martin PE, et al. Ten Kilometer performance and predicted velocity at VO2max among well-trained male runners. Med Sci Sports Exerc. 1989;21(1):78–83. doi: 10.1249/00005768-198902000-00014 2927305

[31] Wang TY, Chan KH, Hsu MC, et al. Effects of consecutive 7-day high-versus moderate-intensity training on endurance determinants and muscle damage in basketball players. Int SportMed J. 2012;13(1):18–28.

[32] Martins C, Kazakova I, Ludviksen M, et al. High-intensity interval training and isocaloric moderate-intensity continuous training result in similar improvements in body composition and fitness in obese individuals. Int J Sport Nutr Exerc Metab. 2016 Jun;26(3):197–204. doi: 10.1123/ijsnem.2015-0078 26479856

[33] Batacan RB Jr., Duncan MJ, Dalbo VJ, et al. Effects of high-intensity interval training on cardiometabolic health: a systematic review and meta-analysis of intervention studies. Br J Sports Med. 2017 Mar;51(6):494–503. doi: 10.1136/bjsports-2015-095841 27797726

[34] Kostic AD, Howitt MR, Garrett WS. Exploring host-microbiota interactions in animal models and humans. Genes Dev. [2013 Apr 1];27(7):701–718. doi: 10.1101/gad.212522.112 23592793 PMC3639412

[35] Cresci GA, Bawden E. Gut microbiome: what we do and don’t know. Nutr Clin Pract. 2015 Dec;30(6):734–746. doi: 10.1177/0884533615609899 26449893 PMC4838018

[36] Hasan N, Yang H. Factors affecting the composition of the gut microbiota, and its modulation. PeerJ. 2019;7:e7502. doi: 10.7717/peerj.7502 31440436 PMC6699480

[37] Stevenson JL, Krishnan S, Stoner MA, et al. Effects of exercise during the holiday season on changes in body weight, body composition and blood pressure. Eur J Clin Nutr. 2013 Sep;67(9):944–949. doi: 10.1038/ejcn.2013.98 23695203

[38] Tomasik PJ, Sztefko K, Pizon M. The effect of short-term cold and hot exposure on total plasma ghrelin concentrations in humans. Horm Metab Res. 2005 Mar;37(3):189–190. doi: 10.1055/s-2005-861296 15824975

[39] Crabtree DR, Blannin AK. Effects of exercise in the cold on ghrelin, PYY, and food intake in overweight adults. Med Sci Sports Exerc. 2015 Jan;47(1):49–57. doi: 10.1249/MSS.0000000000000391 24870575

[40] Mitranun W, Deerochanawong C, Tanaka H, et al. Continuous vs interval training on glycemic control and macro- and microvascular reactivity in type 2 diabetic patients. Scand Med Sci Sports. 2014;24(2):e69–e76. doi: 10.1111/sms.12112 24102912

[41] Schaun GZ, Pinto SS, Silva MR, et al. Whole-body high-intensity interval training induce similar cardiorespiratory adaptations compared with traditional high-intensity interval training and moderate-intensity continuous training in healthy men. The J Strength & Cond Res. 2018;32(10):2730–2742. doi: 10.1519/JSC.0000000000002594 29746386

[42] Yatsunenko T, Rey FE, Manary MJ, et al. Human gut microbiome viewed across age and geography. Nature. [2012 May 9];486(7402):222–227. doi: 10.1038/nature11053 22699611 PMC3376388

[43] Saunders PU, Pyne DB, Telford RD, et al. Factors affecting running economy in trained distance runners. Sports Med. 2004;34(7):465–485. doi: 10.2165/00007256-200434070-00005 15233599

[44] Clark A, Mach N. The crosstalk between the gut microbiota and mitochondria during exercise. Front Physiol. 2017;8:319. doi: 10.3389/fphys.2017.00319 28579962 PMC5437217

[45] American Diabetes Association Professional Practice C. 2. Classification and diagnosis of diabetes: standards of medical care in diabetes-2022. Diabetes Care. [2022 Jan 1];45(Suppl 1):S17–S38. doi: 10.2337/dc22-S002 34964875

[46] Pugh JN, Impey SG, Doran DA, et al. Acute high-intensity interval running increases markers of gastrointestinal damage and permeability but not gastrointestinal symptoms. Appl Physiol Nutr Metab. 2017 Sep;42(9):941–947. doi: 10.1139/apnm-2016-0646 28511020

[47] Antunes BM, Campos EZ, Santos RVTD, et al. Anti-inflammatory response to acute exercise is related with intensity and physical fitness. J Cell Biochem. 2019 Apr;120(4):5333–5342. doi: 10.1002/jcb.27810 30324630

[48] Liu H, Li W, Wang X, et al. Early gut mucosal dysfunction in patients with acute pancreatitis. Pancreas. 2008;36(2):192–196. doi: 10.1097/MPA.0b013e31815a399f 18376312

[49] Shen L, Ao L, Xu H, et al. Poor short-term glycemic control in patients with type 2 diabetes impairs the intestinal mucosal barrier: a prospective, single-center, observational study. BMC Endocr Disord. [2019 Mar 8];19(1):29. doi: 10.1186/s12902-019-0354-7 30849982 PMC6408809

[50] Hung YL, Suzuki K. The pattern recognition receptors and lipopolysaccharides (lps)-induced systemic inflammation. Int J Res Stud Med Health Sci. 2017;2:1–7.

[51] Al-Attas OS, Al-Daghri NM, Al-Rubeaan K, et al. Changes in endotoxin levels in T2DM subjects on anti-diabetic therapies. Cardiovasc Diabetol. [2009 Apr 15];8(1):20. doi: 10.1186/1475-2840-8-20 19368716 PMC2674418

[52] Karczewski J, Troost FJ, Konings I, et al. Regulation of human epithelial tight junction proteins by lactobacillus plantarum in vivo and protective effects on the epithelial barrier. Am J Physiol Gastrointest Liver Physiol. 2010 Jun;298(6):G851–9. doi: 10.1152/ajpgi.00327.2009 20224007

[53] Mumford SL, Dasharathy S, Pollack AZ, et al. Variations in lipid levels according to menstrual cycle phase: clinical implications. Clin Lipidol. [2011 Apr 1];6(2):225–234. doi: 10.2217/clp.11.9 21743815 PMC3130301

[54] Matthews KA, Crawford SL, Chae CU, et al. Are changes in cardiovascular disease risk factors in midlife women due to chronological aging or to the menopausal transition? J Am Coll Cardiol. [2009 Dec 15];54(25):2366–2373. doi: 10.1016/j.jacc.2009.10.009 20082925 PMC2856606

